# *SALL1* expression in acute myeloid leukemia

**DOI:** 10.18632/oncotarget.23448

**Published:** 2017-12-15

**Authors:** Huda Salman, Xiao Shuai, Anh Thu Nguyen-Lefebvre, Banabihari Giri, Mingqiang Ren, Michael Rauchman, Lynn Robbins, Wei Hou, Hasan Korkaya, Yupo Ma

**Affiliations:** ^1^ Georgia Regent University Cancer Center, Augusta, GA, USA; ^2^ Present address: Stony Brook University Cancer Center, Stony Brook, NY, USA; ^3^ Department of Hematology, West China hospital of Sichuan University, Chengdu, P.R. China; ^4^ Department of Nephrology, Saint Louis University, St Louis, MO, USA

**Keywords:** SALL1, AML

## Abstract

Similar signaling pathways could operate in both normal hematopoietic stem and progenitor cells (HSPCs) and leukemia stem cells (LSCs). Thus, targeting LSCs signaling without substantial toxicities to normal HSPCs remains challenging. SALL1, is a member of the transcriptional network that regulates stem cell pluripotency, and lacks significant expression in most adult tissues, including normal bone marrow (NBM). We examined the expression and functional characterization of SALL1 in NBM and in acute myeloid leukemia (AML) using *in vitro* and *in vivo* assays. We showed that SALL1 is expressed preferentially in LSCs- enriched CD34+CD38- cell subpopulation but not in NBM. SALL1 inhibition resulted in decreased cellular proliferation and in inferior AML engraftment in NSG mice and it was also associated with upregulation of PTEN and downregulation of m-TOR, β-catenin, and NF-қB expression. These findings suggest that SALL1 inhibition interrupts leukemogenesis. Further studies to validate SALL1 as a potential biomarker for minimal residual disease (MRD) and to determine SALL1’s role in prognostication are ongoing. Additionally, pre-clinical evaluation of SALL1 as a therapeutic target in AML is warranted.

## INTRODUCTION

Acute myeloid leukemia (AML) is characterized by excessive expansion of myeloblasts in the bone marrow. Regardless of their morphologic subtypes, only a fraction of these cells can recapitulate leukemia generation in sublethally irradiated NOD/SCID mice [1]^.^ These leukemia generating cells, the LSCs, can arise from the neoplastic transformation of normal HSPCs [2, 3]. Like normal HSPCs, LSCs are enriched in the CD34^+^CD38^-^ cell subpopulation [4, 5]^.^ Immunophenotype alone is not reliable to accurately define LSCs. LSCs have been defined as CD34^+^CD38^-^CD90^-^CD71^-^, in contrast to HSPCs which have a phenotype most consistent with CD34^+^CD38^-^CD90^+^CD71^+^ [^5]^. But still, LSCs may also be CD90^+^ or even CD34^-^ [6, 7]^.^ Furthermore, LSCs are not successfully targeted or particularly sensitive to current chemotherapy or immunotherapy regimens because they are mostly quiescent, express multidrug resistant genes (MDRs), and are likely less immunogenic than more mature progenitors [8]. Because they share essential cellular pathways with normal HSPCs like *Bmi1, PTEN* and quiescence genes [9, 10]. one major challenge in targeting LSCs is identifying pathways that are not required for normal HSPC survival and differentiation to minimize detrimental hematopoietic toxicity.

*Sall1* is one of four members of the *Spalt* (‘Spalt-like’ (Sall/HSAL)) family of evolutionarily conserved genes that were originally identified in *Drosophila* and are critical for organogenesis [11]. *SALL1*, is zinc-finger transcription factor and a member of the transcriptional network that regulates embryonic stem cell pluripotency, in association with Nanog and Sox2 [12]. It has a unique expression profile in adult tissues, which includes the kidneys and brain but it is not significantly expressed in normal marrow [13, 14]. Mice lacking *Sall1* expression are not viable as a result of a severe kidney dysgenesis [15]. In humans, heterozygous mutations of *SALL1* can lead to Townes-Brocks syndrome, an autosomal dominant developmental disorder that is characterized by kidney and heart anomalies together with other phenotypic abnormalities [16]. *SALL1* silencing via promoter hypermethylation was described in human breast cancer [17]^.^ and lymphoid leukemia [18]. On the other hand *SALL1* is highly expressed in Wilm’s tumors and is associated with aggressive behavior suggesting dual oncogenic and tumor suppressor function [19, 20]. More recently *Sall1* was reported to regulate an inflammatory program in the otherwise ‘immunologically unique central nervous system via maintaining microglia as resting tissue macrophages rather than proinflammatory phagocytes. *Sall1* deletion in microglia produced up- regulation of genes encoding proinflammatory proteins and activation of genes associated with the identity of other tissue macrophages [21]. This suggests a role for *SALL1* in immune regulation at least in the brain. Two *SALL1* transcript variants encoding different protein isoforms (isoforms A and B) have been reported (NCBI data base).

Here we report on the constitutive expression of *SALL1* in human AML and its potential role in AML development. Most importantly, we present *SALL1* as a potential unique molecular marker for LSCs.

## RESULTS

### SALL1 is expressed in human primary AML samples and in AML cell lines but not significantly in NBM samples

**Figure 1:**
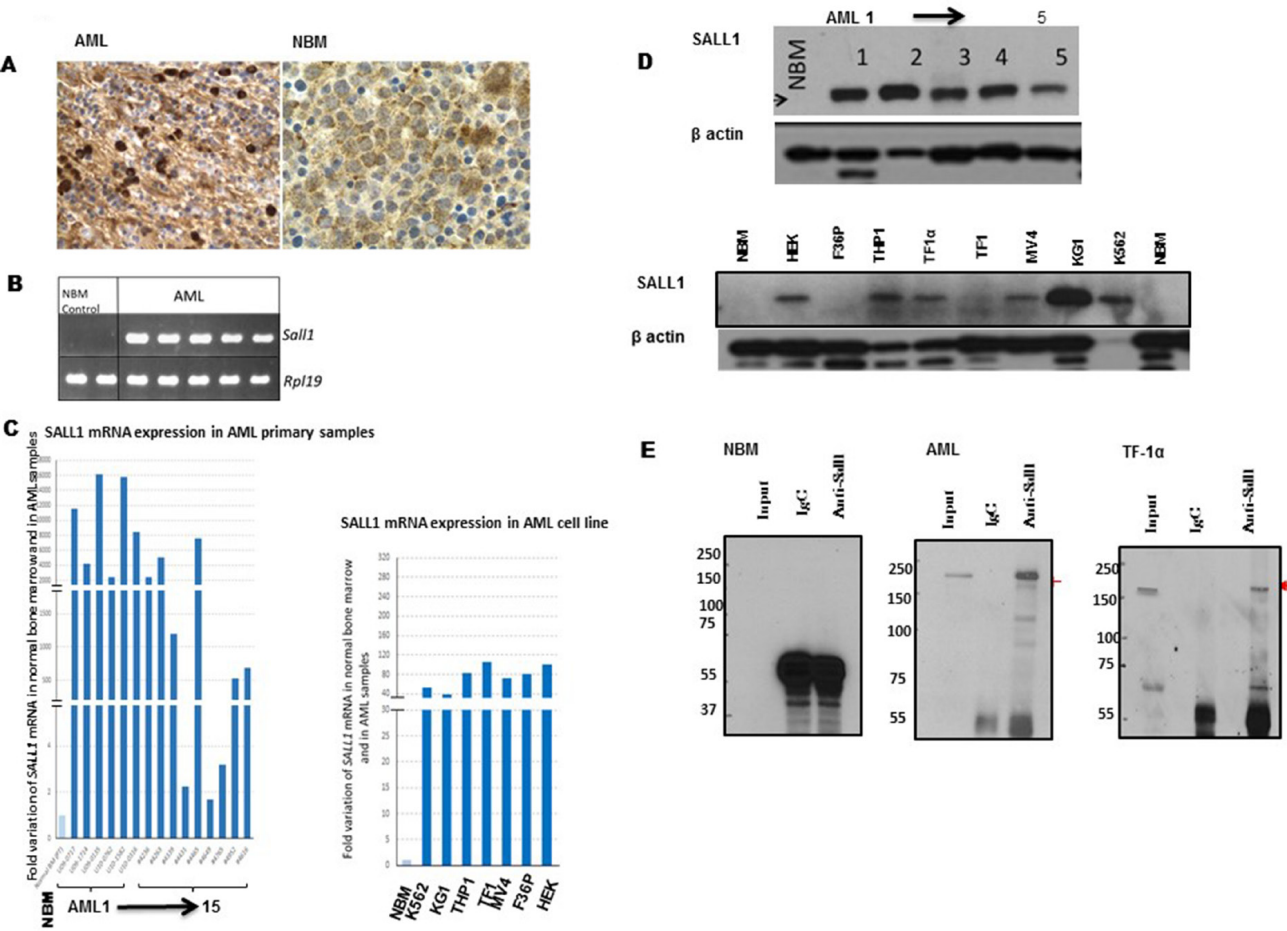
SALL1 is expressed in human AML samples and cell lines (**A**) Positive nuclear IHC staining for SALL1 in AML compared to NBM. (**B**) RT-PCR and (**C**) qRT-PCR analysis of SALL1 expression in AML samples and AML cell lines as well as in NBM samples. The expression data were normalized to Rpl19 standard invariant gene levels. (**D**) Western blotting analyses of SALL1 protein expression in AML samples, and cell lines and in NBM sample. (**E**). Immunoprecipitation of RIPA buffer harvested total protein. Proteins were then analyzed by SDS-PAGE and membranes probed with monoclonal anti-*SALL1* antibodies.

Expression assay by immunohistochemistry revealed positive nuclear *SALL1* immunostaining in 50 randomly selected AML samples, Figure 1A. The genetic characterization of these samples are shown in Table 1. Positive staining was defined as > 15% of cells demonstrating a nuclear staining. In the majority of the cases, more than 30% of the cells were positive, and there was a strong correlation between *SALL1* positivity and blast count. In occasional cases, weak cytoplasmic staining was observed, however, this was regarded as nonspecific as only definitive nuclear staining was considered to be positive. None of the ten NBM samples showed any *SALL1* expression by immunostaining.

**Table 1:** Genetic characteristics of AML samples

Normal karyotype (NO = 25)	Monosomy 7 (NO = 10)	Monosomy 8 (NO = 3)	CBF t(8;21) (NO = 7)	inv16 (NO = 3)	Unknown (NO = 2)
FLT3-ITD: 7 IDH2 mutation:3	FLT3-ITD: 3			FLT3-TKD: (1 with inv16)	

Reverse transcription (RT)-PCR, Figure 1B, and quantitative reverse transcription Q-RT-PCR, Figure 1C were used to evaluate mRNA expression patterns of *SALL1* in AML primary samples (15 shown, 50 examined), AML cell lines (*n* = 7) and in NBM (2 shown, 10 examined). HEK293 cells were used as positive control. *SALL1* mRNA was expressed in leukemia cells and cell lines at a much higher level than in NBM. Relative expression of *SALL1* is illustrated in relation to ribosomal protein gene *RPL19* expression as a housekeeping gene. There was significant inter-sample variation of *SALL1* m-RNA level compared to normal marrow expression, ranging from 2- to 16000-fold. This wide variation was not observed across AML cell lines in which expression levels ranged between 40-to 110-fold compared to NBM.

We next examined *SALL1* protein patern of expression by WB. AML samples (5 shown, 20 examined) and AML cell lines (No = 7) were tested. *SALL1* is reported to be expressed at two sizes, a ∼150 kDa and a ∼200 kDa protein bands, possibly reflecting the two variants of coding mRNA (A: NM_002968.2 and B: NM_001127892.1). In our study, these two variants were both expressed in HEK293 cells. In contrast, one *SALL1* protein band was detecetd in AML patients’ samples and in the majority of AML cell lines, while no *SALL1* protein was detected in NBM, Figure 1D. Immunoprecipitation confirmed that *SALL1* is undetectable in NBM, Figure 1E. Furthermore, *SALL1* peptides were isolated from the corresponding band stained by 1% Coomassie brilliant blue and analysed by mass spectrometry, Supplementary Figure 1. Unlike the specific *SALL1* expression in AML and AML cell lines, other *SALL* family proteins, *SALL2* and *SALL4* were also expressed in NBM, Supplementary Figure 2.

### *SALL1* is preferentially expressed in LSCs enriched CD34+/CD38- cell subpopulation in AML

**Figure 2:**
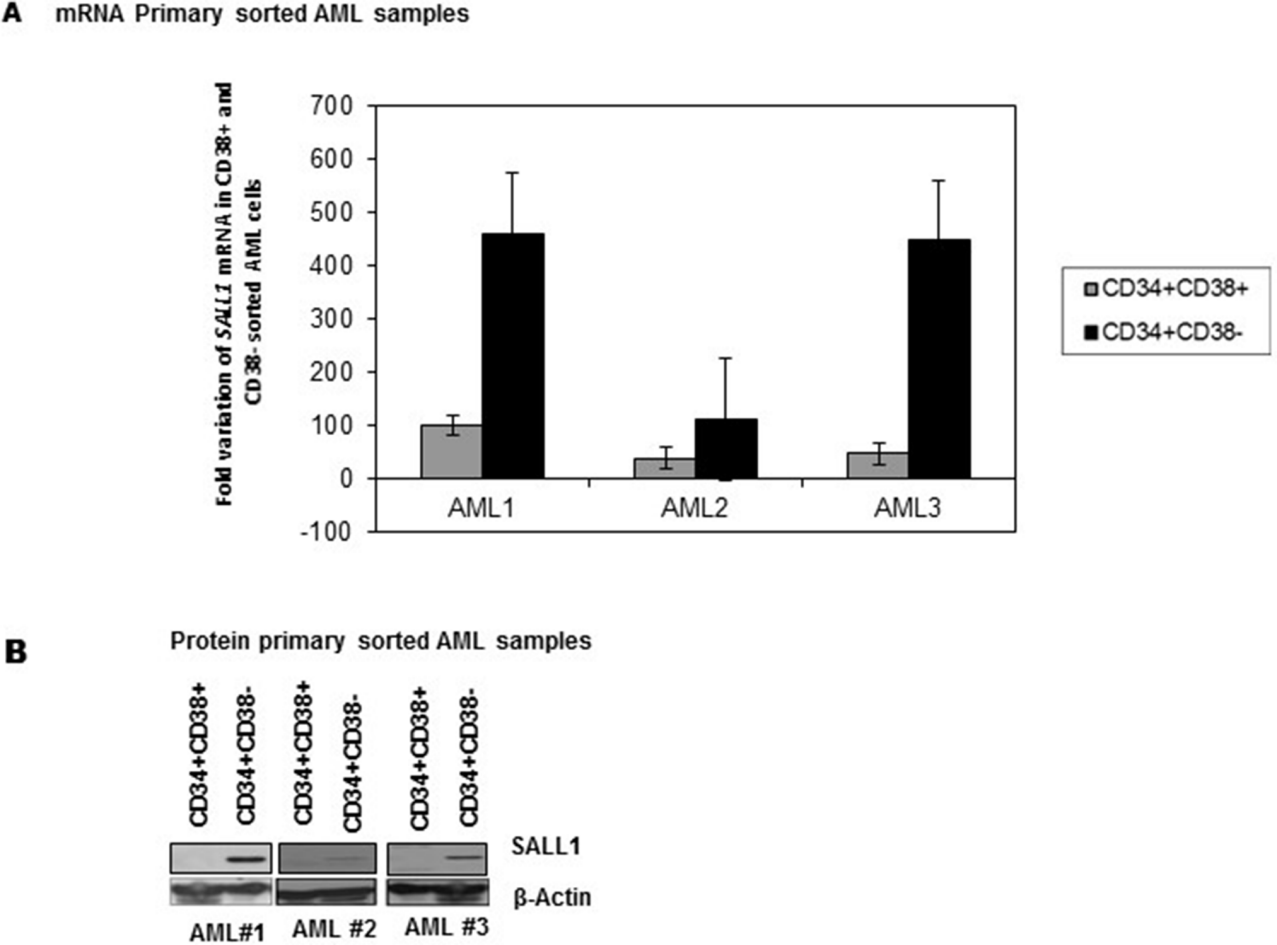
Preferential SALL1 expression in LSCs enriched CD34+/CD38- cell subpopulation. (**A**) qRT-PCR analysis of SALL1 mRNA expression in sorted LSCs enriched CD34+/CD38- subpopulation compared to CD34+/CD38+ subpopulation (**B**). Western blotting analysis of SALL1 protein expression in sorted AML cells.

*SALL1* expression has been associated with stem cell signature patterns [30]. LSCs enriched CD34+/CD38- cell subpopulation preferentially expressed *SALL1* mRNA, Figure 2A, and SALL1 protein, Figure 2B. SALL1 was not detected in NBM.

### SALL1 exonal pattern and protein size in AML indicate the presence of a novel protein isoform

**Figure 3:**
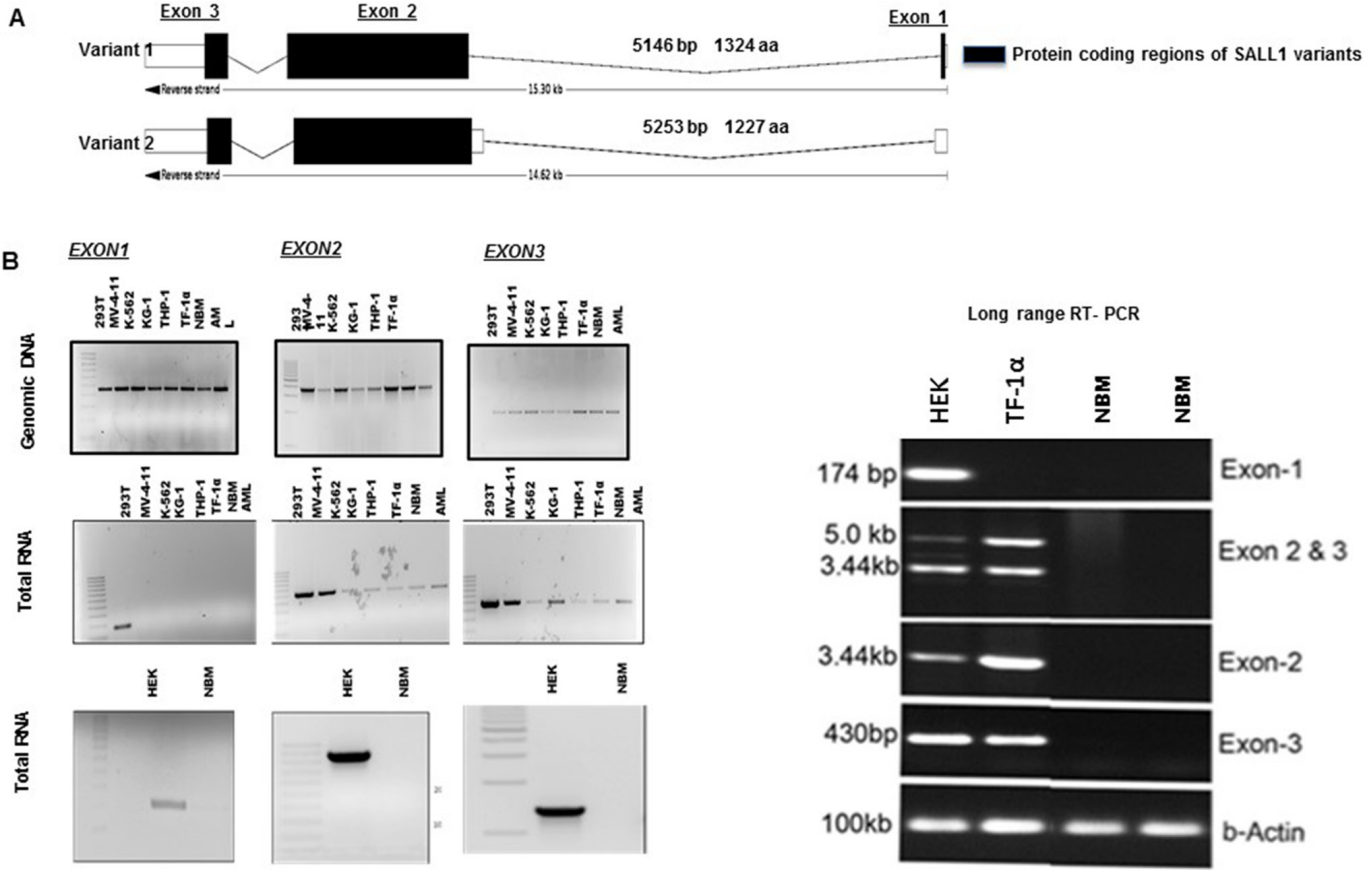
Exonal pattern of SALL1 expression on AML (**A**) Schematic illustration of SALL1 gene: All three exons of variant 1 are protein coding, while only exons 2 and 3 are in variant 2. (**B**). PCR of SALL1 genomic DNA and RT-PCR of *SALL1* mRNA expression in HEK293, AML cells, AML cell lines and in NBM: All three exons of *SALL1* are detected in genomic DNA of HEK293, AML cell lines, NBM, and AML. cDNA from *SALL1* exon1 was expressed in HEK293 cells, but not in AML cells and cell lines. Exons 2 and 3 were detected in HEK293 cells and malignant cells. *SALL1* mRNA was not detected in NBM. (**C**). Long-range RT-PCR using exon specific primers: exons 1, 2 and 3 were detected HEK293. Exons 2 and 3 but not exon1 were detected in AML TF1-α cell line at one length. *SALL1* was not detected in NBM. Our primer is designed to detect the 3’ end of exon 2.

Four transcripts of protein coding *SALL1* are reported with NM_001127892.1 (isoform B) and NM_002968.2 (isoform A) being the most studied. Protein coding regions of transcript variant 1 and variant 2 are illustrated in Figure 3A (dark sections). To determine which *SALL1* transcript is activated in AML, we first examined the presence of each of *SALL1’s* three exons. Both genomic DNA and cDNA were examined, Figure 3B. All three exons of *SALL1* genomic DNA were detected in HEK293T, primary AML, and AML cell lines and in NBM alike, first panel Figure 3B. Reverse transcriptase was used to determine mRNA expression. All three exons were expressed in HEK293 cells while exon 1 was not detected in transcripts from AML and AML cell lines. We further verified this expression pattern with long range PCR. HEK293T cells expressed the three *SALL1* mRNA encoding exons as well as exons 2 and 3 in one transcript. Only transcripts expressing exons 2 and 3 were confirmed to exist in AML cells and cell lines. *SALL1* mRNA expression was not detected in NBM. The absence of exon 1 expression in AML suggested that *SALL1* transcript variant 2 (NM_001127892.1) is the activated transcript in AML. However the first peptide that was detected by mass spectrometry sequence GQPSRPTK, Supplementary Figure 1, is in fact encoded in the presence of transcript 1 exon 2 cDNA and not transcript 2. To explain this finding, we examined the *SALL1* transcripts as reported in Ensembl genome browser 90. As reported, in transcript 1 (NM_002968.2) translation starts at the beginning of exon 1 (ATG 017-020) and continues through the 5′ of exon 2 and deep into exon 3. In transcript 2 (NM_001127892.1) translation begins deeper in exon 2 (ATG 433-435) missing the beginning of exon 2 3′. Consistent with the primer location that worked best to detect SALL1 (5′ GAGACACAGAAAAGGGTCAACCG 3′- 108–130), we believe that translation in AML starts at the beginning of exon 2 (ATG 105–107) of transcript variant 1. This will account for a protein that is missing the first 25aa of isoform 1, and explain the *SALL1* band size in AML that is close to the 200Kd band reported for isoform 1 protein. This also is supported by that our data clearly show that exon1 mRNA is not detected in AML.

### SALL1 inhibition results in inferior cell proliferation and inferior AML engraftment in mice

**Figure 4:**
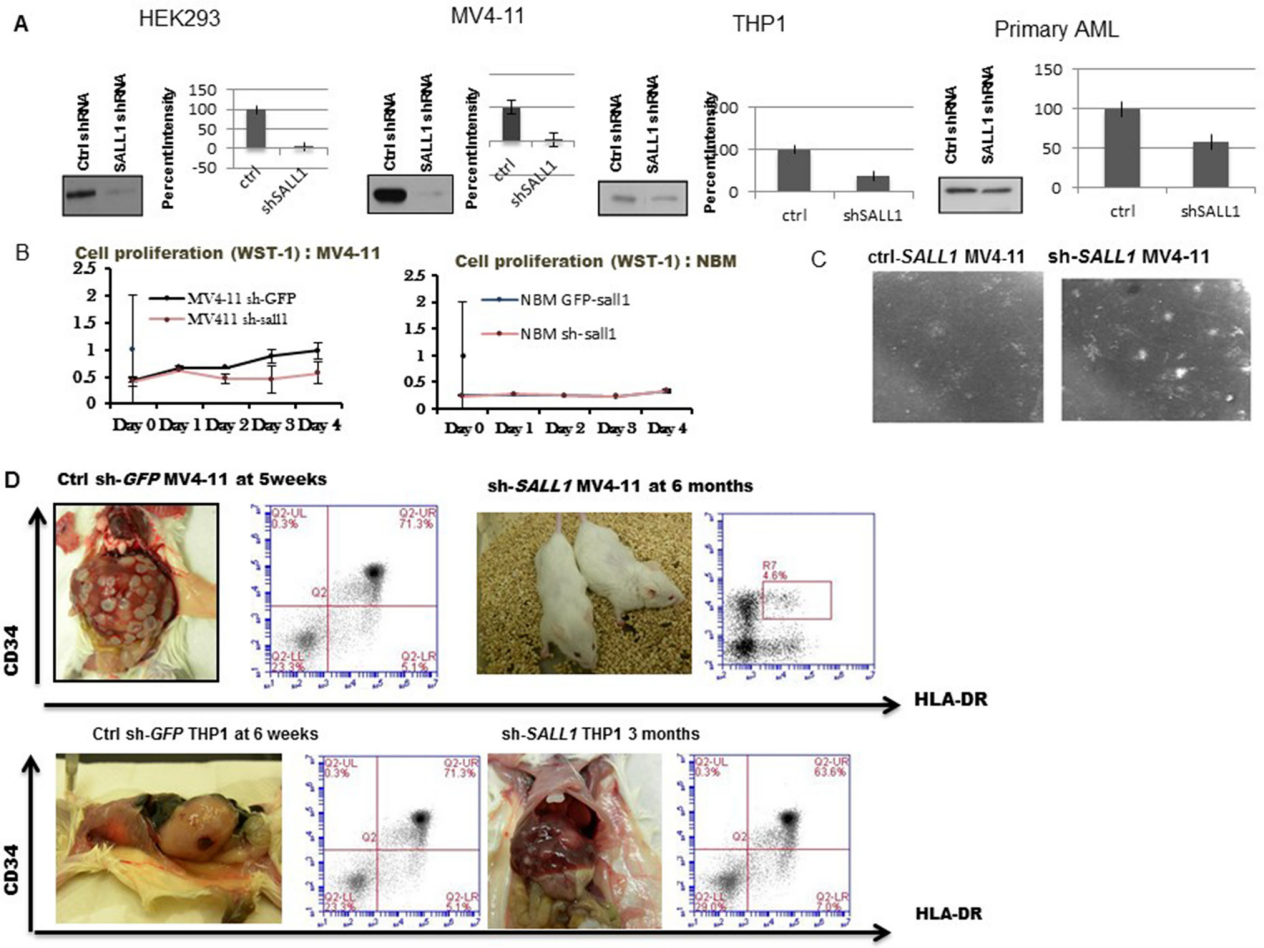
SALL1 Inhibition is associated with decreased cellular proliferation, improved GM-CFUs formation and inferior AML engraftment in NSG mice (**A**) qRT-PCR and western blot analysis of *SALL1* inhibition: HEK293 cells, 2 AML cell lines (MV4-11 and THP1) were stably infected and 1 AML primary patient sample was transiently infected with shRNAs of SALL1. Transfection efficiency was different amongst different cells ranging between >90% in MV4-11 to 40% in AML primary cells. (**B**) Cell proliferation: MV4-11 cells treated with sh-RNA against *SALL1* or against GFP were incubated with cell proliferation WST-1 reagent. Sh-*SALL1* treated cells showed a significant reduction in proliferation. And in (**C**) induced GM-colony formation. (**D**) In vivo engraftment of sh-SALL1 and of ctr-GFP MV4-11 and THP1 cells: SALL1 inhibition in MV4-11 cells resulted in AML engraftment and mice became morbid at an average of 5 weeks compared to mice injected with GFP-SALL1 MV4-11 cells which were alive and looked healthy at 6 months after treatment and with no evidence of leukemia when sacrificed. Spleen cells are sorted to HLA-DR and CD34 human AML markers. Less efficient SALL1 inhibition in THP1 cells resulted in delayed AML engraftment by an average of 9 weeks.

To determine the effects of *SALL1* inhibition on cellular proliferation and leukemia engraftment in mice, HEK293 cells, AML cell lines MV4-11 and THP1cells were stably infected with either sh-RNA constructs (NO 4) directed against *SALL1* (*sh-SALL1*) or against GFP (ctrl-GFP-*SALL1*) with over 80% transfection efficiency. Primary AML CD34+ positive cells and NBM cells were transiently infected without puromycin selection with either sh-RNA against *SALL1* or against GFP. These sh-RNA constructs were aligned with *SALL1* variants sequence to confirm specificity. SALL1 protein expression and residual target mRNA levels following *SALL1* inhibition were determined via WB and q RT–PCR, Figure 4A. *SALL1* expression was downregulated the most, by over 90% in HEK293 cells, and in MV4-11 AML cell lines, while 60% level of expression reduction was achieved in THP-1 cell lines and only modestly in primary AML samples. NBM CD34 + cells were treated as a control; no expression (or downregulation) was detected and shRNA treatment did not affect these cells (see proliferation assay, Figure 4B). *sh-SALL1* MV4-11 cells demonstrated inferior proliferation when treated with Proliferation Reagent WST-1 compared to ctrl-GFP-*SALL1* MV4-11 cells. No difference was seen in HEK293 proliferation with *SALL1* inhibition (data not shown) . NBM CD34+ cells showed similar proliferation in both *sh-SALL1* treated and ctrl-GFP-*SALL1* treated plates. Of note, proliferation difference was more apparent in MV4-11 cells that have less residual *SALL1* than THP-1(data not shown). This may indicate that *SALL1* affects cellular proliferation in a dose dependent manner, although experiments involved two different cell lines. *SALL1* inhibition was associted with improved granulocytic and monocytic colony forming unit capacity (GM-CFUs) compared to ctrl-GFP-*SALL1* treated cells , Figure 4C, suggesting that loss of SALL1 lead to differentiation of leukemic cells. GM-CFUs were identified by morphology.

To examine the effect of *SALL1* downregulation on leukemia engraftment *in vivo*, we traced human AML cell lines engraftment and leukemia progression in irradiated NSG mice injected with MV4-11 or THP1 cells treated with either *sh-SALL1* or ctrl-GFP-*SALL1*, 5 mice in each group, Figure 4D. Human cells engraftment was documented in all mice via flowcytometric analysis for (GFP^+^)/CD45^+^. Mice engrafted with MV4-11 ctrl-GFP-*SALL1* treated cells developed AML and died on an average of 5 weeks, significantly earlier than those engrafted with MV4-11 sh-*SALL1* cells. The latter remained alive and showed no symptoms of sickness at 6 months with no experiment related death in this mice cohort and a *p* value of < 0.01 based on a non parametric Wilcoxon rank test. In the THP1 group, mice engrafted with ctrl-GFP-*SALL1* THP1 cells developed AML and died at an average of 6 weeks, about 9 weeks earlier compared to those injected with *sh-SALL1* THP1 who developed AML and died at average of 16 weeks with a *p* value 0.0122. Again *SALL1* inhibition in THP1 was not as efficient as it was with MV4-11 (∼60% downregulation versus 90%). When sacrificed after 6 months, sh-*SALL1* MV4-11 were found to be engrafted with CD45+ human cells yet they remained leukemia free.

### SALL1 inhibition was associated with alterations in cellular protein expression including PTEN upregulation

**Figure 5:**
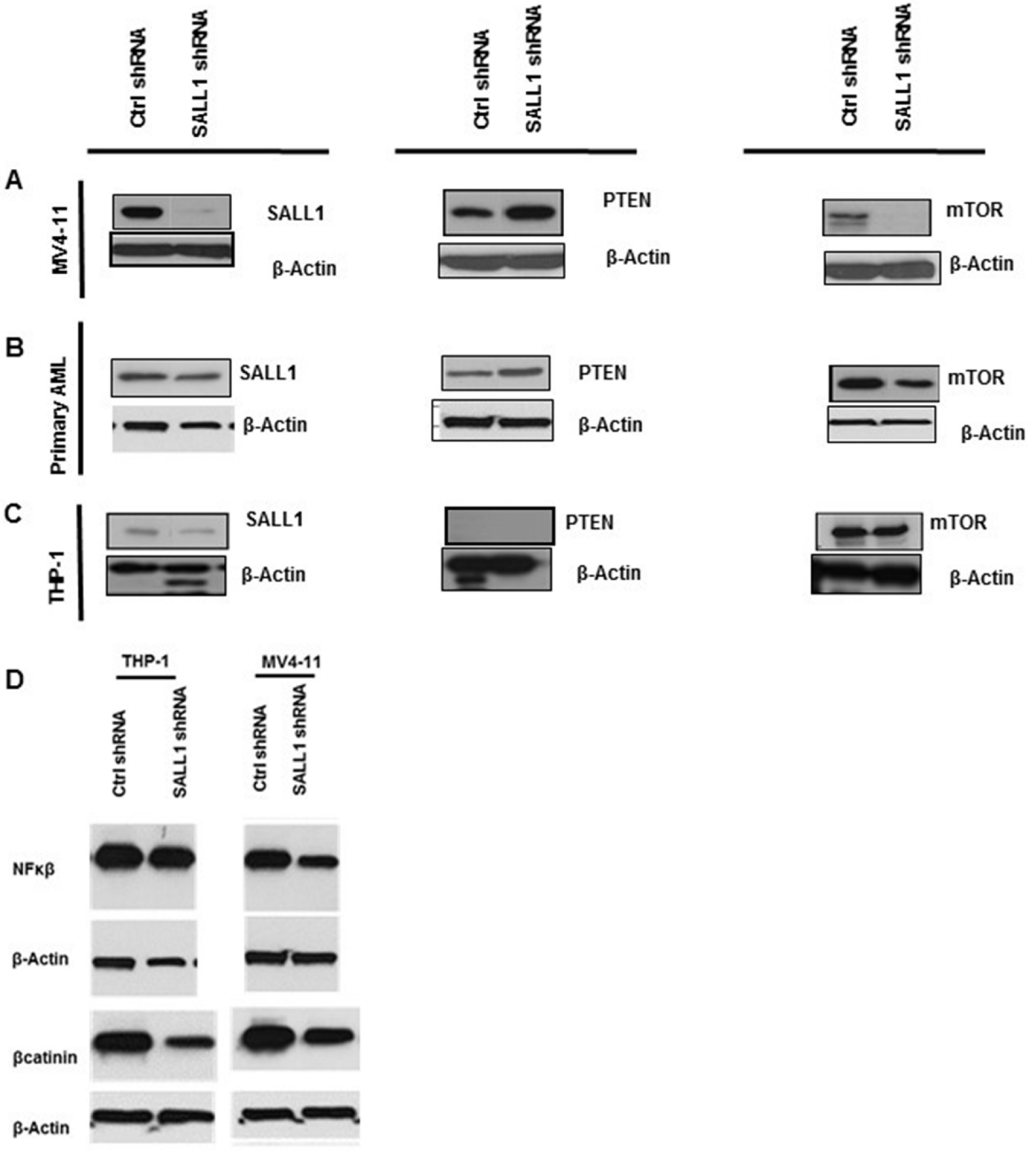
SALL1 inhibition resulted in upregulation of PTEN expression and inhibition of down-stream m-TOR and in downregulation of B-catenin and NF-κB: (**A, B**) SALL1 inhibition resulted in upregulation of PTEN and inhibition of m-TOR in MV4-11 and primary AML, more prominent with MV4-11 where SALL1 downregulation efficiency was almost 100%, than in primary AML. (**C**) In genomic *PTEN* deleted THP1 cells, mTOR did not change. (**D**) *SALL1* inhibition is shown with decreased B-catinin, and NF-κB expression in in both MV4-11 and THP1.

There is only limitted information about *SALL1*’s role in cellular signaling and transcriptional regulation apart from the few described in embryonic development and in breast and renal cell cancers [17, 18]. To learn about its protein interactions in AML, we hypothesized that *SALL1* may interact with proteins or interfere with signaling pathways in LSC differentiation arrest and proliferation. The tumor suppressor gene phosphatase and tensin homolog (*PTEN*) plays an important role in the balance between proliferation and differentiation of blood cells progenitors. In 2006, two independent studies reported that *PTEN* deletion in HSPCs leads to their lineage fate determination restriction, exhaustion and, importantly, to leukemogenesis [22, 23]. *SALL1* and *PTEN* were reported to have direct interactions with *SALL4* promotors and possibly inter-regulating effects [24, 25]. Thus, we evaluated *PTEN* expression in AML samples and cell lines with and without *SALL1* inhibition. As shown in Figures 5A and 5B, *SALL1*-inhibition resulted in *PTEN* protein upregulation in AML cell lines and primary samples. This was accompanied by downregulation of total and phosphorylated m*-TOR*. In the THP1 cell line, where *PTEN* is reported to be deleted at the genomic leve [26], *SALL1* inhibition did not affect total or phospho-m-TOR, Figure 5C. It however downregulated both *NF-κB*, which is aberrantly expressed in AML progenitor cells, and β-catenin, which exerts control on AML blast proliferation and engraftment [27, 28]. Figure 5D. β catenin was previously reported to be regulated by *SALL1* [29]. β catenin *and NF-κB* were also downregulated when *SALL1* was inhibitied in MV4-11.

## DISCUSSION

The important role of *SALL1*, a homeotic gene and a transcription factor, in human development was recognized because heterozygous *SALL1* mutations lead to Townes-Brocks syndrome. *SALL1′s* oncogenic role in leukemogenesis is described here for the first time.

Biochemical cellular operations vital survival of normal HSPCs and LSCs are mostly similar. Oncogenic alterations confer self-renewing potential by affecting pathways that are also functional in normal stem cells [30]. Hence, targeting such common pathways is often limited by significant toxicity to normal cells. Pursuing genes, proteins or signaling pathways involved in leukemogenesis, but with little or no involvement in normal HPCs survival and differentiation would be ideal for therapeutic target development [10, 31]. We identified a *SALL1* isoform that is expressed in AML, and preferentially in the LSCs enriched CD34+/CD38- subpopulation, but not in NBM. Reports on normal marrow expression of *SALL1* amongst available data sets such as the human protein atlas (http://www.proteinatlas.org/search/sall1) and uniProt (http://www.uniprot.org/uniprot/Q9NSC2) are inconsistant. The former reports one case displaying a weak cytoplasmic and membranous staining for *SALL1* in NBM and the latter excludes NBM from *SALL1* tissue expression. In this study we showed that *SALL1* protein is not expressed in NBM And that it is expression pattern in AML is strictly nuclear. These findings demonstrate that it is possible to identify transcriptional circuits and biomarkers that are unique to neoplastic LSCs and not functionally required in their normal HSPCs counterparts within the exact same tissue. *SALL1* distinct expression in LSCs and lack of expression in HSPCs suggests that *SALL1* is a potential unique biomarker for AML. *SALL1* expression is not limited to a subset of AML patients . Moreover, it is not a mutation or an overexpression above a cetrtain baselin in NBM, but rather *de novo* distinctive expression in LSCs. Such a decisive pattern of a biomarker expression is direly needed in the MRD setting in the absence of another AML specific phenotypic or molecular marker, which occurs in 40% of AMLs. Verification study that *SALL1* could offer this important distinction in this critical setting is ongoing.

*SALL1* inhibition conferred weaker proliferation on AML cells and AML cell lines. We and others have previously reported that loss of *Sall1* in developing kidney leads to ectopic or accelerated differentiation of multipotent renal progenitor cells [32]. Here we show that consistent with its role as a differentiation repressor, *SALL1* inhibition in AML cells promoted them towards differentiation by forming more mature monocytic and granulocytic colonies upon morphological exam of CFU assay. Further studies are necessary to test the differentiation advantage of *SALL1* inhibition *in vivo.*

To directly test the impact that *SALL1* loss has on leukemogenic *in vivo*, we generated a humanized mouse xenograft using human AML cell lines with shRNA *SALL1* inhibition or sh-GFP. We showed that *SALL1* inhibition was associated with inferior leukemia engraftment and prolonged survival compared to GFP- xenografted control cells. Our investigation of the potential mechanism of *SALL1* involvement in leukemogenesis demonstrated that *SALL1* inhibition restored or increased *PTEN* expression in AML cells and cell lines. Leukemogenesis is sensitive to even subtle changes in *PTEN* dosage. Consequently, mechanisms regulating *PTEN* protein expression could potentially play a critical role in leukemia development and possibly its course, response to treatment and prognosis [22]. Thus restoring *PTEN* functions directly or indirectly holds potential therapeutic promise. Inhibition of *SALL1* may potentially be an indirect way to upregulate *PTEN* as a therapeutic goal. *SALL1* inhibition did not upregulate *PTEN* levels “further” in HEK293 cells in which *PTEN* was abundant with and without *SALL1* inhibition. Of note, *PTEN* also plays an important role in balancing HSCs normal proliferation and differentiation [10, 22]. Additionally our xenograft model suggests that other leukemogenic pathways such as β-catenin and *NF- қB* were also impacetd by the loss of *SALL1,* particularly interesting in THP1 cells (*PTEN*-deleted).

In ongoing work, the potential for the clinical application of *SALL1* as a molecular marker for monitoring of MRD in AML as compared to other standard methods will be validated. Concurrently, we will examine if *SALL1* expression level according to a standerized expression score on IHC and on a standarized fold-expression assessment by RT PCR is of prognostic value that could impact treatment outcomes. Additionally, our ongoing work will examine if *SALL1* inhibition *in vivo* preclinical models can lay foundations for future clinical therapeutic targetting.

## MATERIALS AND METHODS

### Cell cultures, lentiviral infections, mRNAi, cell proliferation and CFU colony formation assay

Embryonic kidney cell line HEK293T, known to express *SALL1*, and AML cell lines (MV4-11, THP-1,KG-1, K-562, TF-1, TF-1α) as well as CD34+ cells sorted from normal BM were purchased from ATCC ( American Type Culture Collection, Manassas, VA, USA). HEK293T cell line was grown at 37°C with 5% CO2 in Dulbecco’s modified Eagle medium (DMEM from Gibco/Life Technologies, Carlsbad, CA, USA) and used as positive control for *SALL1* expression. AML cell lines were grown at 37°C with 10% CO2 in RPMI 1640 (Gibco/Life Technologies), both supplemented with 10% fetal bovine serum (ATLANTA biologicals, Inc., Flowery Branch, GA, USA) and 50 U/mL penicillin and 50 µg/mL streptomycin (Gibco/Life Technologies). Patients’ primary AML cells were provided as a courtesy of Dr. *Guido Marcucci* (Ohio State University, Columbus, OH, USA) and by the Georgia Regents University Tumor Bank (Augusta, GA, USA). Primary AML cells were cultured in serum free conditions in StemSpanTM SFEM (STEMCELL Technologies, Vancouver, BC, V5Z 1B3, Canada).

Primary cells and cell lines were infected with a lentiviral supernatant (≥ 5 × 10^6^ CFU/μL) produced in HEK293 cells (Thermo Scientific The RNAi Consortium Lentiviral shRNA and Thermo Scientific Trans-Lentiviral Packaging Kits) with a Dharmacon™ shRNA or ORF transfer plasmid. A pool of 4 specific 29mer target-specific shRNA, and one scramble control (OriGene) directed against human *SALL1* mRNA or GFP were used to stably infect HEK293 cells, 2 AML cell lines (MV4-11 and THP1). 1 AML primary patient sample and NBM CD34 positive cells (ATCC) were transiently infected with the *SALL1* shRNAs for 72 hours and then selected for 4 days in the presence of 2 µg/mL and 1.5 µg/mL puromycin (ThermoFisher Scientific, Waltham, MA, USA). The sequences of the 4 constructs are: AGCGAAGCCTCAACATTTCCAATCCGACC, CTCAAGGTACTTTACGAACATCTGCCAAC, CCAGCCATCTCAGAGTCTACCTCTTCCAT and TTGCTTGTCAGAGTGCCTTGGACATTCAC and all aligned with *SALL1* variants of interest sequence.

Cell proliferation assay was performed with the Cell Proliferation Reagent WST-1 following the manufacturer’s instructions (Roche, San Francisco, CA, USA). Cells were seeded in 96 well plate (5,000 cells in 90 µL of culture medium/well) for different time points. Cells were incubated in presence of 10 µL of WST-1 reagent for 1 h before measuring the absorbance of the samples at 450 nm. For colony formation assay, MV4-11 Cells treated with sh-*SALL1* or ctrl-GFP-*SALL1* were suspended in RPMI and diluted into a final volume of 400 µL RPMI. The final 400 µL cell suspensions were added to 4.0 mL of MethoCult H4320 and plated in triplicate and incubated at 37°C/5% CO2 in a humidified environment for 10 d prior to counting. Only colonies with ≥ 50 cells were counted and analyzed by morphology.

### Extraction of total proteins and SDS--polyacrylamide gel electrophoresis (SDS-PAGE) western blot and immunoprecipitation analysis

Cells were harvested in lysis buffer containing 20 mM Tris-HCL pH 7.5, 150 mM NaCl, 1 mM EDTA, 1 mM EGTA, 1% Triton X-100 or RIPA, 2.5 mM Na4P2O7 (ThermoFisher Scientific) and 1% protease and phosphatase inhibitor cocktail (Roche). Concentration of total proteins was determined with Bio-Rad Protein Assay (Bio-Rad, Hercules, CA, USA).

Different quantities of proteins per sample were analyzed by SDS-PAGE and membranes were probed with anti-*SALL1* antibody (1:1000; Abcam, Cambridge, MA, USA, #ab41974), anti-*PTEN* antibody (1:2000; Cell Signaling Technology,Danvers, MA, USA, #9559), anti-mTOR antibody (1:1000; Cell Signaling Technology, #2972), anti-phosphorylated-mTOR antibody (1:5000; Abcam, #ab109268), anti-NF-κB antibody (1:1000; Cell Signaling Technology, #3987), anti-phosphorylated-NF-κB antibody (1:1000; Cell Signaling Technology, #3031), anti-TRIB2 antibody (1:1000; Cell Signaling Technology, #13533) and anti-β-Actin-Peroxidase antibody (1:50,000; Sigma-Aldrich, Saint-Louis, MO, USA, #A3854). Proteins were incubated with primary antibodies overnight at 4°C. Secondary HRP-conjugated mouse or rabbit (both from Thermo Fisher Scientific, #31432 and #31462 respectively) antibodies were used at a dilution of 1:5000. Membranes were developed using the SuperSignal^®^ West Femto Maximum Sensitivity Substrate (Thermo Fisher Scientific, #34094). Protein quantification was performed with the Image J software (Research Services Branch (RBS), Image Processing and Analysis in Java (http://rsb.info.nih.gov)). For immunoprecipitation, total proteins were extracted using the RIPA buffer containing 50 mM Tris-HCl pH 7.4, 1% Nonidet-P40, 0.5% C24H39NaO4 (sodium deoxycholate), 0.1% SDS, 150 mM NaCl, 2mM EDTA, 50 mM NaF (all from Thermo Fisher Scientific) and 1% protease and phosphatase inhibitor cocktail (Roche). Two micrograms of total proteins were incubated overnight, at 4°C with either 15 µL anti-*SALL1* antibody (Abiocode, Agoura Hills, CA, USA, #R1428-1) or 2 µg anti-Rabbit IgG antibody (Rockland, Gilbertsville, PA, USA, #KCC003). The next day, 50 µL of Protein A/G coupled to magnetic beads (Thermo Fisher Scientific) were added to total proteins for 3 h at 4°C. Total proteins were then submitted for magnetic sorting, following the manufacturer’s instructions. Proteins associated to the beads were resuspended into 80 µL of 2X Laemmli buffer, containing 50 mM Tris-HCl pH 6.8, 2% SDS, 0.1% Bromophenol blue, 100 mM Dithiothreitol (DTT) and 10% glycerol. Proteins were then analyzed by SDS-PAGE and membranes were probed with anti-*SALL1* antibody (Abcam).

### Preparation of total proteins for SDS-PAGE and mass spectrometry (MS)

Total proteins from KG1 leukemic cell line were extracted as described above and separated by SDS-PAGE in 8% polyacrylamide gel. Proteins were stained by 1% Coomassie brilliant blue G250 (Sigma-Aldrich) in 1% phosphoric acid, 5% ammonium sulfate and 8% ethanol. De-staining of the slab gel was achieved with distilled water. Pieces of gel containing proteins of interest were cut out and sent to Applied Biomics, Inc, Proteomics Service (Hayward, CA, USA) for mass spectrometry and protein/peptides identification. Gel pieces were then treated and proteins contained in gel pieces were digested with trypsin. Peptides were extracted and MALDI-TOF/TOF MS analysis was performed. Protein identification was based on peptide fingerprint mass mapping using mass spectrometry data and on peptide fragmentation mapping using MS/MS data. The peaklist files were searched against the UniProtKB/Swiss-Prot or NCBI database using MASCOT search engine to identify proteins.

### Extraction of genomic DNA, total RNA, RT-PCR and quantitative-PCR

Total gDNA was prepared from HEK293T, leukemic cell lines and one AML sample from patient with the DNAzol^®^ Reagent (Gibco / Life Technologies) following the manufacturer’s instructions. *SALL1* exons were amplified by PCR with specific primers: exon 1 was amplified with the forward primer 5′GCTCGATTTCCGTAATTTTGAG 3′ and the reverse primer 5′ AAAGCAGGGACGGAGAAGCC 3′; exon 2 was amplified with the forward primer 5′ TACAGTTTTGAGTTTTGCAGGTG 3′ and the reverse primer 5′ TTCCCTTGCAAGAGCTCTCTC 3′; and exon 3 was amplified with the forward primer 5′ GTTGCCTTCACTGTCTGTCAC 3′ and the reverse primer 5′ GGAGTAGGAGGCCACCATAG 3′. PCR products were analyzed on agarose gel and bands containing the DNA of interest were cut out. DNA was extracted from agarose gel with the MinElute Gel Extraction Kit (Qiagen) following the manufacturer’s instructions. Concentration of the total amount of gDNA was determined by measuring the absorbance at 260 nm with a NanoDrop spectrophotometer (Thermo Fisher Scientific). Purified PCR products were send to ACGT, INC (Wheeling, IL, USA) for single pass DNA Sequencing.

Total RNA was extracted with the RNeasy Mini Kit (Qiagen, Hilden, Germany), followed by a DNAse treatment (Ambion, Austin, TX, USA). Concentration of the total amount of RNA was determined by measuring the absorbance at 260 nm with a NanoDrop spectrophotometer (Thermo Fisher Scientific). Reverse transcription (RT) assays were performed on 3 µg of total RNA with the SuperScript™ III Reverse Transcriptase (Invitrogen/Life Technologies). PCR experiments were performed with the Platinum^®^ Taq DNA Polymerase High Fidelity (Invitrogen/Life Technologies) and analyzed by Bio-Rad C1000™ Thermal Cycler (Bio-Rad). Quantitative PCR experiments were performed with the iQ™ SYBR^®^ Green Supermix (Bio-Rad) and analyzed by Bio-Rad CFX96™ Real-time System (Bio-Rad).

Primers for qRT-PCR: forward sequence: 5′GAGACACAGAAAAGGGTCAACCG 3′ and reverse sequence: 5′ TTCTCGATGATGACGTTGCTG 3′. Standard invariant gene used for quantification of *SALL1* total mRNA was ribosomal protein L19 (RPL19; UniProtKB accession number: P84098). For each pair of primers, the efficiency of qRT-PCR was determined using a standard curve generated by a dilution series of the sample that had the highest expression rate for the selected gene. The global quantification for each gene was based on the published mathematical formula 2.

### *In vivo* studies

We chose two cell lines for our *in vivo* studies. MV4-11 and THP1 AML cell lines treated with either *shSALL1* or ctrl-GFP-*SALL1* were injected in the tail vein of irradiated Nonobese diabetic/severe combined immunodeficiency/interleukin (IL) 2R^null^ (NSG) mice. Each mouse was injected with 1.5 x10^6^ cells, 5 mice in each group. Mice were evaluated daily for morbidity, weight loss, failure to thrive, and splenomegaly. Peripheral blood (PB) was obtained from the retro-orbital venous plexus and samples were analyzed periodically for green fluorescent protein (GFP^+^)/CD45^+^ human cells engraftment and for leukemia generation by flowcytometry. Premorbid animals were sacrificed by CO_2_ asphyxiation, and then hematopoietic tissues were removed for subsequent analysis.

The relevant institutional review boards approved the research.

## SUPPLEMENTARY MATERIALS FIGURES AND TABLE



## Abbreviations

AML: Acute Myeloid Lekemia
HSPCs: Hematopoietic Stem and Progenitor Cells
LCSs: Leukemia Stem Cells
NBM: Normal Bone Marrow
GM-CFUs: Granulocytic and Monocytic Colony Forming Unit capacity
RT-PCR: Reverse Transcriptase Polymerase Chain Reaction
qPCR: Quantitative Polymerase Chain Reaction
WB: Western Blot
IHC: Immunohisochemestry
ctrl: Contro/
GFP: Green Florescent Protein
