# Supplementary material for: SALL1 expression in acute myeloid leukemia
Source: Oncotarget. 2017 Dec 15;9(7):7442–52. doi: 10.18632/oncotarget.23448 (PMC5800914; doi:10.18632/oncotarget.23448)
Supplement: Supplementary file 1 [file oncotarget-09-7442-s001.pdf]

SALL1 expression in acute myeloid leukemia

SUPPLEMENTARY MATERIALS

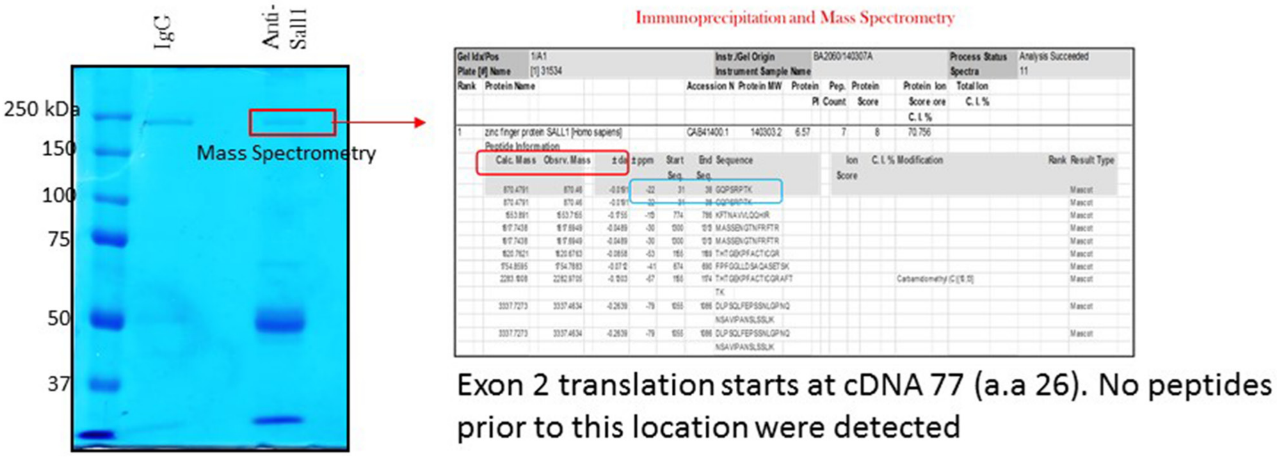

Supplementary Figure 1: Massspecrometry: Total proteins separated by SDS-PAGE in 8 % polyacrylamide gel and stained by 1 % Coomassie brilliant blue and gel containing proteins of interest were cut out for mass spectrometry. SALL1 specific peptides were identified. Of note, Exon 1 encoded peptides were not identified.

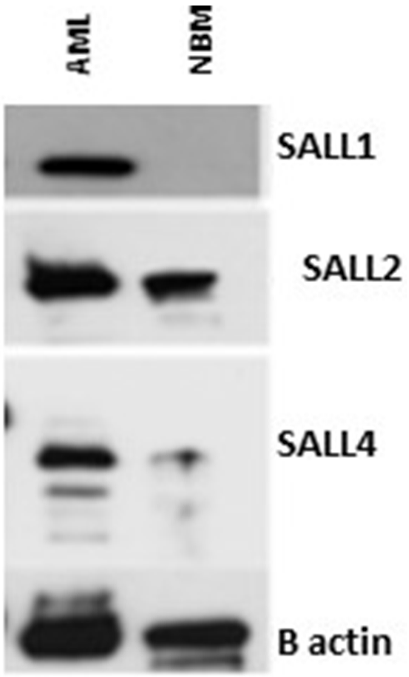

Supplementary Figure 2: Other SALL protein family members are expressed in NBM and in AML: SALL1 expression is exclusive to AML and lacks in NBM. SALL4 and SALL2 are expressed in both but may be overexpressed in AML.
